# Anatomical Description of a Chinese Foot

**Published:** 1830-04-01

**Authors:** 


### Anatomical Description of a Chinese Female Foot. By Bransby Cooper, Esq.

The *penchant* of the most ancient people of the world for little feet is well known, and the *pains* taken by the Chinese fair to please their liege lords in this particular have been blabbed abroad by the tell-tale pens of curious oriental travellers. Some twelve months back, one of that active class of individuals who live on the love of novelty possessed by the most gullible public of Europe, imagined that he should make his fortune by exhibiting two poor Chinese damsels in the modern Babylon. They were accordingly incarcerated for a season in a little room in Pall-Mall and astonished a few of the British belles, but whether or not the speculation answered is more than we pretend to know. We are not aware that any anatomical description of the Chinese female foot is on record, and are therefore tempted to take notice of one by Mr. Bransby Cooper, in the Philosophical Transactions for the present year. It is of course as a matter of curiosity, and that only, but matters of curiosity are no mean articles of consumption in the present day.

The body from which the foot now in abeyance, as the lawyers term it, was taken, belonged to a female found floating in the river at Canton. The subject being from the lower order of persons, art had probably not done so much in producing deformity or beauty, be it which it may, as it accomplishes in the higher classes of Chinese. Such is our want of taste that Mr. Cooper remarks, "we should either at first sight consider it as that species of deformity vulgarly called club-foot or the result of some accidental dislocation, which from ignorance and want of surgical skill, had been left unreduced." With what supreme contempt would an aristocratic mandarin listen to such an exposé of *mauvais ton* as this!

"From the heel to the great toe the foot is unusually short, not exceeding five inches, and is said in some instances to measure even less than this; and the great toe itself, which in its natural and free state projects in a straight-forward direction, is bent with a peculiar abruptness upwards and backwards, whilst the remaining toes with the exception of the first phalanx of the second and third, are doubled in beneath the sole of the foot so as to leave scarely any breadth at this part of the foot, which in the unconstrained limb is commonly the broadest; and the striking shortness of the heel scarcely projecting beyond the line of the leg, which itself descends upon the foot at a considerable obliquity from behind forwards, imparts an appearance to the foot as if it were kept in a state of permanent extension. The upper surface of the foot is very convex; but its convexity is irregular and unnatural, presenting a sudden and prominent projection just anterior to the external malleolus, and above the outer extremity of a deep cleft which traverses the sole of the foot." It is in this latter part, the sole, that the most remarkable alterations are observed, and accordingly our able dissector passes at once to its description.

### SOLE OF THE FOOT.

Supposing the body placed upon its back and the foot resting on the heel, the great toe is bent backwards towards the leg, and the second toe is so twisted under it that its extremity reaches the inner edge of the foot. The two extreme phalanges of the third toe are placed more obliquely than the second and consequently do not reach so far inwards, but all the phalanges of the two remaining toes are bent under the plantar region of the foot in such a manner as to produce a visible depression in its external edge. Exterior and posterior to the nail of the third toe is a corn, another large corn in nearly the same situation on the fourth, and two more corns upon the fifth. The little toe twisted inwards across the sole forms the anterior boundary to a deep cleft or hollow, extending transversely across the whole breadth of the foot between the toes and the heel. "To judge from its appearance, one might suppose that the heel and toes had been forcibly brought together so as considerably to diminish the whole length of the foot, and to convert its natural longitudinal hollow into that deep concavity. The heel which forms the other boundary of the cleft presents a large square surface, if not entirely flattened, yet with a striking diminution of convexity, so as to suggest the probability that it affords the principal point of support in progression; a surmise which is further corroborated by the great density of the skin at this part."

### DORSUM OF THE FOOT.

The appearance of this is completely altered also; the dorsum rising with an unusual convexity not only from behind forwards, but also from side to side. It affords a distinct protuberance just before the external malleolus, and above the outer extremity of the cleft in the sole, which is here very conspicuous; anterior to this eminence, the dorsum presents a plane surface facing outwards, till it slopes off rapidly beneath where the toes are turned under the sole. At the junction of the great toe with the dorsum there is a considerable angle, resulting from the dent or hollow which the abrupt direction of the great toe upwards and forwards produces upon that surface. From this dorsal point of view the two last toes are not seen, they being buried beneath the foot. The only thing worth remarking posteriorly is the extreme shortness of the heel.

"The integuments covering the heel are unusually dense, hard, and resisting, and the cuticle is of a remarkable thickness. The subcutaneous structure resembles rather the fatty sole of a horse's foot than any human tissue. The skin which covers the rest of the sole presents a corrugated appearance and is somewhat thicker than in an ordinary foot: but in those places where it had been defended from external pressure by the intervention of the toes, which passed under it, it does not deviate from the natural construction." On the dorsum the integuments offer nothing unusual, and the only circumstance to be noted is the particular convexity of the nail of the great toe from side to side. The tendons are merely altered in direction, but the state of the bones is very striking.

### SKELETON OF THE FOOT.

"The position of the os calcis is very remarkably altered: instead of the posterior projection of the heel, a straight line is preserved in this direction, not deviating from the line of the tibia; and the projecting point which forms in an ordinary foot the most posterior process, and into which the tendo achillis is inserted, touches the ground and becomes the point d'appui for sustaining the whole weight of the body. The articular surface of the calcis and cuboid bone is about half an inch anterior to, and two inches above this point, while the astragalar joint is behind and somewhat below the calco-cuboidal articulation; consequently the long axis of the os calcis, instead of being from behind forwards, is from below upwards, with the slightest possible inclination forwards. The most prominent points of the instep are the round head of the astragalus, and the cuboidal articulation of the os calcis. From this the remaining tarsal bones slope downwards at nearly a right angular inclination to join the metatarsal bones, whose obliquity is still downwards until they rest on their phalangeal extremities."

The length between the os calcis where it touches the ground and the most anterior part of the metatarsal bone of the great toe, is four inches;—that of the foot, including the toes, 6¼ inches;—height of the instep 3½ inches. Thus the arch of the foot has a span of two inches and a quarter with a height of two inches, which space is filled up with the condensed cellular substance before described. The cleft of the foot is three inches in depth, the greatest width of the foot barely two inches. The points of support are the os calcis, the anterior extremity of the metatarsal bone of the great toe, and the dorsal surface of the fourth and fifth toes, which are bent under the foot so as to press the ground at this part.

Such are the anatomical particulars of this singular deformity, and we are sure that the scientific members of the profession will be much obliged to Mr. Cooper for the trouble he has taken in ascertaining and detailing them.

